# Estrogen and/or Estrogen Receptor α Inhibits BNIP3-Induced Apoptosis and Autophagy in H9c2 Cardiomyoblast Cells

**DOI:** 10.3390/ijms19051298

**Published:** 2018-04-26

**Authors:** Bih-Cheng Chen, Yi-Jiun Weng, Marthandam Asokan Shibu, Chien-Kuo Han, Yueh-Sheng Chen, Chia-Yao Shen, Yueh-Min Lin, Vijaya Padma Viswanadha, Hsin-Yueh Liang, Chih-Yang Huang

**Affiliations:** 1 School of Post-Baccalaureate Chinese Medicine, China Medical University, Taichung 404, Taiwan; cbc@mail.cmu.edu.tw; 2 Graduate Institute of Basic Medical Science, China Medical University, Taichung 404, Taiwan; juweng2014@gmail.com (Y.-J.W.); shibu.m.a@gmail.com (M.A.S.); 3 Department of Health and Nutrition Biotechnology, Asia University, Taichung 404, Taiwan; jackhan@asia.edu.tw; 4 School of Chinese Medicine, China Medical University, Taichung 413, Taiwan; yuehsc@mail.cmu.edu.tw; 5 Department of Nursing, MeiHo University, Pingtung 912, Taiwan; x00003061@mail.meiho.edu.tw; 6 Department of Pathology, Changhua Christian Hospital, Changhua 500, Taiwan; 93668@cch.org.tw; 7 Department of Medical Technology, Jen-Teh Junior College of Medicine, Nursing and Management College, Taipei 11260, Taiwan; 8 Department of Biotechnology, Bharathiar University, Coimbatore 641 046, India; padma.vijaya@gmail.com; 9 Graduate Institute of Clinical Medical Science, China Medical University, Taichung 404, Taiwan; liangsy2@gmail.com; 10 Division of Cardiology, China Medical University Hospital, Taichung 404, Taiwan; 11 Department of Biological Science and Technology, Asia University, Taichung 404, Taiwan

**Keywords:** BNIP3, estrogen receptor alpha, apoptosis, autophagy

## Abstract

The process of autophagy in heart cells maintains homeostasis during cellular stress such as hypoxia by removing aggregated proteins and damaged organelles and thereby protects the heart during the times of starvation and ischemia. However, autophagy can lead to substantial cell death under certain circumstances. BCL2/adenovirus E1B 19 kDa protein-interacting protein 3 (BNIP3), a hypoxia-induced marker, has been shown to induce both autophagy and apoptosis. A BNIP3-docked organelle, e.g., mitochondria, also determines whether autophagy or apoptosis will take place. Estrogen (E2) and estrogen receptor (ER) alpha (ERα) have been shown to protect the heart against mitochondria-dependent apoptosis. The aim of the present study is to investigate the mechanisms by which ERα regulates BNIP3-induced apoptosis and autophagy, which is associated with hypoxic injury, in cardiomyoblast cells. An in vitro model to mimic hypoxic injury in the heart by engineering H9c2 cardiomyoblast cells to overexpress BNIP3 was established. Further, the effects of E2 and ERα in BNIP3-induced apoptosis and autophagy were determined in BNIP3 expressing H9c2 cells. Results from TUNEL assay and Immunoflourecense assay for LC3 puncta formation, respectively, revealed that ERα/E2 suppresses BNIP3-induced apoptosis and autophagy. The Western blot analysis showed ERα/E2 decreases the protein levels of caspase 3 (apoptotic marker), Atg5, and LC3-II (autophagic markers). Co-immunoprecipitation of BNIP3 and immunoblotting of Bcl-2 and Rheb showed that ERα reduced the interaction between BNIP3 and Bcl-2 or Rheb. The results confirm that ERα binds to BNIP3 causing a reduction in the levels of functional BNIP3 and thereby inhibits cellular apoptosis and autophagy. In addition, ERα attenuated the activity of the BNIP3 promoter by binding to SP-1 or NFκB sites.

## 1. Introduction

Autophagy is a process of cellular self-degradation that occurs at low basal levels in the heart and facilitates the removal of damaged organelles, cytosolic proteins and pathogens [1]. However, various stressful conditions such as hypoxia may amplify the incidence of cardiac autophagy and destroy cellular homeostasis, leading to unprecedented cell death through excessive self-digestion and apoptosis [1,2]. Under hypoxic stress for instance, elevated hypoxia inducible factor-1α (HIF-1α)-induces the upregulation of Bcl-2/adenovirus E1B 19 kD an interacting protein 3 (BNIP3) that results in mitochondria-dependent apoptosis. BNIP3 is a member of the pro-apoptotic BH3-only subfamily of Bcl-2 family proteins [3,4]. BNIP3 has a BH3 domain that binds to either Bcl-2 or Bcl-XL. Binding of the C-terminal transmembrane domain of BNIP3 to the mitochondrial membrane initiates an apoptotic cascade that results in the depolarization and opening of mitochondrial permeability transition pores (MPTP), a process that leads to mitochondrial dysfunction and subsequent cell death via apoptosis necrosis or autophagy [5,6,7,8,9,10]. BNIP3 has been reported to be the major contributor of cardiac damage under conditions such as ischemia/reperfusion injury by inducing mitochondrial dysfunction [11,12].

Various studies have confirmed the involvement of BNIP3 in cardiac cell death. Higher expressions of BNIP3 in the heart correlate with increased cardiac cell death and the occurrence of cardiac hypertrophy, cardiomyopathy. However, deficiency of BNIP3 expression obstructs the ventricle remodeling process in post-myocardial infarction in mice exposed to ischemia–reperfusion by reducing apoptosis [13,14,15]. Compensative cardiomyocyte hypertrophy is generally the cellular response to adapt to increasing left ventricle wall tension, which involves amplification of protein synthesis. Their adaptive effects last only when the compensatory hypertrophy sustains and on progression to pathological hypertrophy, a maladaptive phenomenon; the resulting cardiac changes result in cardiomyocyte apoptosis followed by fibrosis in the left ventricle causing ventricular chamber stiffness and transition to heart failure. Considering reports from various studies, myocyte hypertrophy and apoptosis may not be even inferred to as opposing effects [16,17,18,19,20,21]. However, autophagy appears to play dual roles in cardiac hypertrophy, and their mechanism in cardiac hypertrophy is complex. The present consensus on cardiac autophagy is that, physiological levels of autophagy are crucial for cellular homeostasis, and any imbalance in the levels of autophagy may lead to cardiomyocyte loss [22]. Basal levels of autophagy are essential to remove damaged or redundant organelles and protein aggregates, and thereby they preserve normal cardiomyocyte survival and function [23]. However, under specific conditions, autophagy in contrary to providing protection against cell death, may in fact mediate cell death. It is important to note that autophagic events and their morphological features are also observed along with the apoptotic events in dying cells [24]. While autophagy triggered by mild stress plays a protective effect by inhibiting apoptosis, excessive stress may cause autophagic events to supplement or even to co-operate with apoptotic cell death [25,26]. Prolonged hypoxia triggers excessive autophagy resulting in cell death, and therefore autophagy is also an appropriate target under specific circumstances to prevent heart disease [27].

Inhibition of cardiomyocyte apoptosis is traditionally an attractive therapeutic approach to protect the heart from associated post-infarction remodeling; however, strategies to inhibit the activation of effectors such as caspase may cause systemic effects, which limits their application [28,29]. Targeting the upstream mediators of apoptosis specific to ischemia-induced cardiomyocyte death is a viable alternative strategy. In this context, Bnip3, which is upregulated in the heart and other tissues via HIF-1α during hypoxia, is an ideal target [13,30,31].

Heart disease is more common in men than in women, but the heart disease risk and incidence increases sharply in women with increasing age [32]. Bhuiyan et al. found that rats that underwent ovariectomy procedures were unable to compensate for hypertrophy and showed deterioration of heart function [33]. Xu et al. reported that suppression of ovarian hormones increased left ventricle LV) remodeling in rats of advanced age; however, the remodeling could be attenuated by estrogen replacement [34]. Many studies have reported that the female sex hormone estrogen (E2) and its receptor ERβ play cytoprotective roles in the heart [35,36,37,38,39,40]. However, since ERα and ERβ are known to undertake opposing effects, it is important to check the effects of ERα in hypoxia-associated damages [41]. ERα has been known to attenuate isoproterenol-induced hypertrophic growth in H9c2 cells by preventing cytosolic calcium accumulation [42]. ERα is also known to attenuate LPS-induced apoptosis through inhibition of tumor necrosis factor-α (TNF-α) expression [43].

In this study, we established an in vitro model to mimic hypoxic injury in the heart by engineering cardiomyoblast cells to overexpress BNIP3. Further, the effects of estrogen and ERα in BNIP3-induced apoptosis and autophagy were verified in BNIP3 expressing H9c2 cardiomyoblasts. The results show that ERα/E2 displays a cytoprotective role by protecting H9c2 cells against BNIP3-induced apoptosis and autophagy.

## 2. Results

### 2.1. B-Cell Lymphoma 2 (BCL2)/Adenovirus E1B 19 kDa (BNIP3) Overexpression Induces Apoptosis in H9c2 Cardiomyoblast Cells

**Figure 1:**
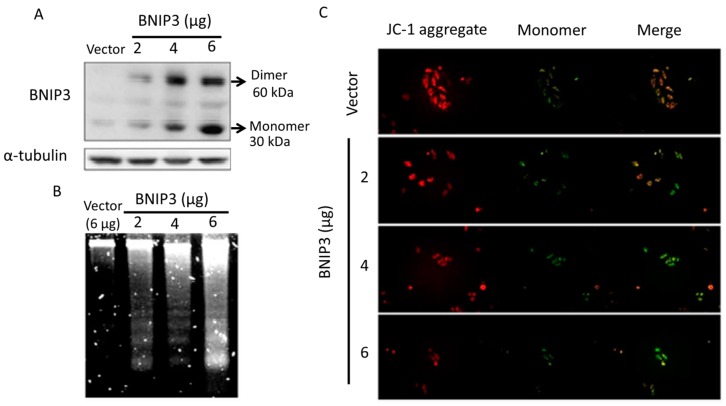
BNIP3 overexpression induces H9c2 cardiomyoblast cell apoptosis. H9c2 cells were transfected with differing amounts (2, 4, 6 μg) of BNIP3-containing plasmids for 24 h. Cells were harvested for different assays. (**A**) Western blot showed that BNIP3 protein expression increased in a dose-dependent manner; (**B**) The degree of DNA fragmentation increased in proportion to the level of B-cell lymphoma 2 (BCL2)/adenovirus E1B 19 kDa (BNIP3) expression; (**C**) JC-1 stain presented mitochondria integrity. The merging of normal (**red**) and damaged (**green**) mitochondria represented the integrity ratio. We found that there was a significant fluorescence emission shift from red to green in cells that overexpressed BNIP3, indicating that BNIP3 overexpression led to mitochondrial damage and DNA fragmentation and, hence, activation of the apoptotic pathway.

Binding of BNIP3 to the mitochondrial membrane destroys the membrane potential and induces mitochondria-dependent apoptosis. To observe whether BNIP3 induces apoptosis, cells were transiently transfected with plasmids (2, 4, 6 µg) containing the full-length BNIP3 gene to overexpress BNIP3 proteins for 24 h. Western blot analysis revealed a dose-dependent increase in the protein level of BNIP3 (Figure 1A). DNA fragmentation, a late-stage apoptotic phenomenon, also increased in proportion with BNIP3 expression (Figure 1B). JC-1 staining is used to detect mitochondrial membrane integrity. Red fluorescence represents aggregation of JC-1 in the mitochondrial intermembrane space, indicating that the mitochondria are intact. In contrast, green fluorescence represents diffusion of JC-1 throughout the cytoplasm, indicating that the mitochondria are damaged. We found that there was a significant fluorescence emission shift from red to green in cells that overexpressed BNIP3 (Figure 1C), indicating that BNIP3 overexpression led to mitochondrial damage, DNA fragmentation, and activation of the apoptotic phenomenon.

### 2.2. Estrogen Receptor α (ERα)/Estrogen (E2) Attenuates the Apoptotic Effect Induced by BNIP3 Overexpression

**Figure 2:**
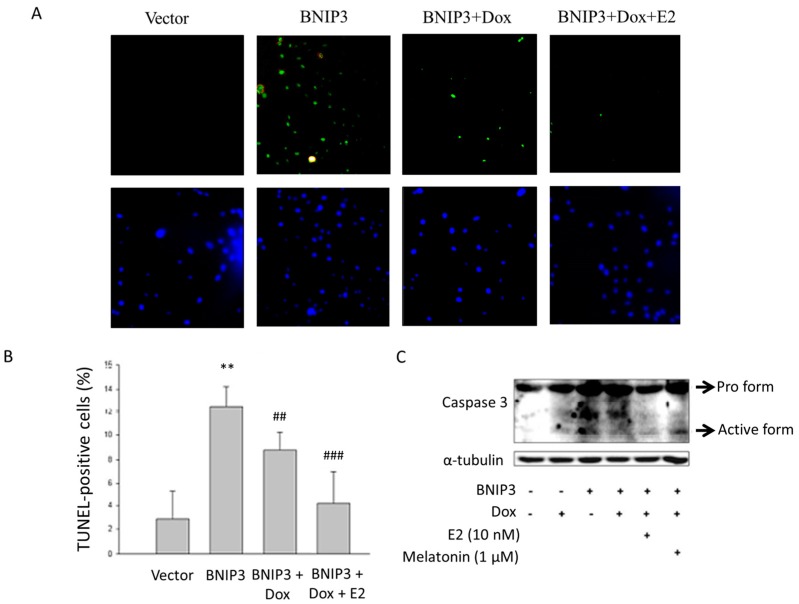
ERα/E2 reversed the apoptotic effect induced by BNIP3 overexpression. Tet-on ERα H9c2 cells were transfected with BNIP3 (6 μg), incubated for 6 h, treated with doxycycline (1 μg/mL) for 1 h, and then exposed to E2 (10 nM) in serum-free medium for 18 h. Cells were fixed and then assayed with the TUNEL test and counter stained with DAPI (blue, nucleus) and 400× microscopic images were taken using a fluorescent microscope. TUNEL-positive cells (green spots) were indicative of dsDNA breaks or ssDNA nicks. (**B**) Quantitative histogram from (**A**). The number of TUNEL-positive cells was significantly greater in BNIP3-transfected cells relative to control (** *p* < 0.01 shows significant difference with respect to control, *n* = 3). The number of TUNEL positive cells in BNIP3-transfected cells that had been exposed to doxycycline (BNIP3+Dox) showed statistical significance versus the number of TUNEL-positive cells in BNIP3-transfected cells (BNIP3) (*^##^ p* < 0.01 and *** *p* < 0.001 show significant differences with respect to BNIP3 group, *n* = 3). (**C**) Tet-on ERα H9c2 cells were transfected with BNIP3 (6 μg), incubated for 6 h, and then exposed to doxycycline (1 μg/mL) or melatonin (1 μM, ERα inhibitor) for 1 h. Cells were then incubated with E2 (10 nM) in serum-free medium for 18 h. Co-treatment of BNIP3-overexpressing cells with doxycycline and melatonin reversed the ERα-related decrease in the level of activated caspase 3 expression. Dox: doxycycline, E2: 17β-estrodiol. *n* = 5.

In order to determine the role of ERα and E2 in regulating the BNIP3 induced apoptosis, BNIP3 plasmids was transfected into Tet-on ERα H9c2 cardiomyoblast cells and were then analyzed the corresponding modulations in the apoptotic process. The TUNEL assay revealed a 9.51% increase in the number of TUNEL-positive cells that overexpressed BNIP3 with respect to the control group (*p* < 0.005). Cells exposed to doxycycline (1 μg/mL) in order to induce ERα, however, showed a 3.78% decrease in the number of TUNEL-positive cells with respect to the BNIP3 group (*p* < 0.05). Further, co-treatment of cells with doxycycline and E2 after BNIP3 transfection resulted in a 4.87% reduction in the number of TUNEL-positive cells (Figure 2A,B). We used Western blot to further examine the protein level of activated caspase 3. The results showed that BNIP3 resulted in an increase in the level of activated caspase 3 expression and a decrease in protein expression after treatment with doxycycline and E2. Co-treatment of BNIP3-overexpressing cells with doxycycline and melatonin, an ERα inhibitor, reversed the ERα-related decrease in the level of activated caspase 3 expression (Figure 2C). ERα protected against BNIP3-induced apoptosis with or without E2 treatment.

### 2.3. ERα/E2 Protects Against BNIP3-Induced Autophagy

**Figure 3:**
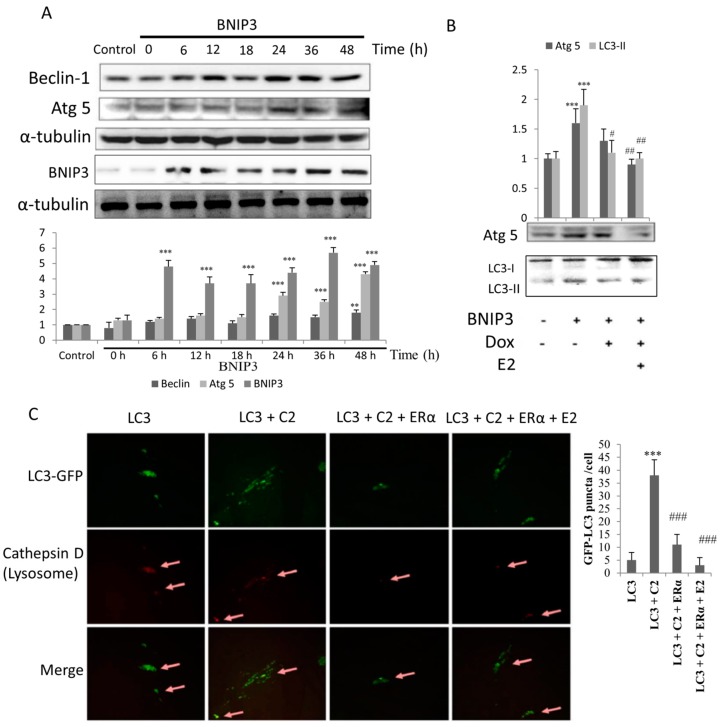
ERα/E2 blocked the BNIP3-induced autophagy effect. H9c2 cells were transfected with BNIP3 (6 μg) and collected at indicated times for Western blot assay. (**A**) The levels of Beclin-1 and Atg 5 increased in proportion to the level of BNIP3 expression. *n* = 5, ** *p* < 0.01 and *** *p* < 0.001 show significant differences with respect to control cells; (**B**) Tet-on ERα H9c2 cells were transfected with BNIP3 (6 μg), incubated for 6 h, and then exposed to doxycycline (1 μg/mL) for 1 h. Cells were then exposed to E2 (10 nM) in serum-free medium for 18 h. Western blot assay was used to detect the levels of proteins involved in the autophagic (Atg5, LC3-I1/LC3-I) pathways. Doxycycline and/or E2 treatment resulted in attenuation of autophagy. *n* = 5, ** *p* < 0.01 and *** *p* < 0.001 show significant differences with respect to the control; ^#^ *p* < 0.05 and ^##^ *p* < 0.01 show significant differences with respect to the BNIP3 group; (**C**) H9c2 cells were transfected with LC3-GFP and/or ERα plasmids and incubated for 6 h, followed by incubation with E2 and/or C2 for 18 h in growth medium. Cells were fixed, incubated with primary anti-cathepsin D antibody (rabbit), and then incubated with secondary anti-rabbit IgG (red). Green fluorescence represents the expression of LC3, and the puncta of green fluorescence represent the active form of LC3 (LC3-II), indicated the formation of autophagosome. Expression of cathepsin D, a lysosome indicator, indicated the location of lysomomes (arrow heads). The merged panels indicate co-localization of autophagosomes and lysosomes (arrow heads). E2: 17β-estrodiol, C2: C2 Ceramide (*N*-Acetyl-d-erythro-Sphingosine, BNIP3 inducer). *n* = 3, *** *p* < 0.001 shows a significant difference with respect to the control LC3 transfected cells group; ^###^ *p* < 0.001 shows a significant difference with respect to the control LC3 + C2 group.

To test whether overexpression of BNIP3 induces autophagy, H9c2 cells were transfected with BNIP3 plasmids and then incubated for 0 to 48 h. We found that the levels of Beclin-1 protein expression gradually increased in a time-dependent manner. In addition, the levels of Atg 5 protein expression increased after 24 h of BNIP3 induction. The results imply that the autophagy pathway was induced by BNIP3 overexpression (Figure 3A). Overexpression of BNIP3 resulted in increased expression of the pro-autophagic proteins Atg5 and LC3-II and that exposure to doxycycline and E2 treatment inhibited the expression of those proteins in Tet-on ERα H9c2 cells. Our results showed that ERα and E2 attenuated the effectors of autophagy in cells that overexpressed BNIP3 (Figure 3B). To further investigate the phenomenon of autophagy, we transfected cells with GFP-tagged LC3 plasmids. The results showed that LC3-GFPs were distributed in cytosol in cells that exhibited a low level of autophagy. Treatment of cells with the BNIP3 inducer C2-ceramide (*N*-Acetyl-d-erythro-Sphingosine) resulted in condensed dots of green fluorescence, which is indicative of cleavage of LC3-GFP to LC3-II-GFP and the subsequent assembly of LC3-II-GFP at the autophagosomal membrane. Cells that had been exposed to ERα with or without E2 treatment presented fewer green dots (Figure 3C, upper panel). In addition, the immunofluorescence assay to detect cathepsin D, a lysosome marker, revealed the location of lysosome. Cells that were transfected with LC3-GFP and C2-ceramide addition showed apparent aggregation of lysosomes and co-localization with autophagosomes (column 2, middle and lower panels). This indicated the formation of autolysosome, the end stage of autophagy. Cells that had been exposed to ERα with or without E2 treatment presented with weaker red fluorescence (column 3 and 4, middle panels). These results suggest that ERα/E2 attenuates the expression of autophagy-related proteins and consequently suppresses the formation of autophagosomes and autolysosomes.

### 2.4. ERα/E2 Blocked Apoptosis and Autophagy by Binding with BNIP3

**Figure 4:**
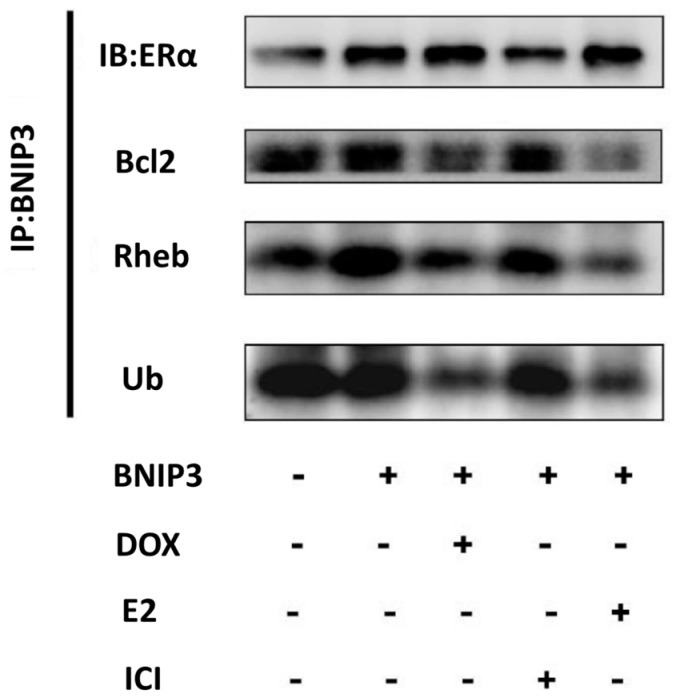
ERα/E2 inhibited apoptosis and autophagy by binding to BNIP3. Tet-on ERα H9c2 cells were transfected with BNIP3 (6 μg), incubated for 6 h, and then exposed to doxycycline (1 μg/mL) or ICI (0.5 μM, ER inhibitor) for 1 h, followed by exposure to E2 (10 nM) in serum-free medium for 18 h. Cell proteins were extracted for immunoprecipitation assay, incubated with primary anti-BNIP3 antibody, and precipitated with protein-G agarose. Immunoblot assay was used to detect the binding of BNIP3 with ERα, Bcl-2 (anti-apoptotic protein), Rheb (anti-autophagic protein), or Ub (ubiquitin, degradation signal protein). E2: 17β-estrodiol, ICI: ICI 182,780.

We found that ERα/E2 inhibited BNIP3-induced cell death; therefore, we investigated how ERα/E2 affects BNIP3. We conducted a co-immunoprecipitation assay to analyze the binding effect of BNIP3 with other proteins. An immunoblot assay revealed increased protein expression of ERα, Bcl-2, Rheb, and ubiquitin in Tet-on ERα-exposed H9c2 cells that had been engineered to overexpress BNIP3. After the addition of doxycycline or E2, however, we found that BNIP3 bound strongly to ERα and weakly to Bcl-2, Rheb, and ubiquitin (Figure 4). The results suggest that ERα competed with Bcl-2 for binding to BNIP3, implying that Bcl-2 exerts an anti-apoptotic effect. Similarly, ERα competed with Rheb for binding to BNIP3, thereby allowing Rheb to activate mTOR, which resulted in the inhibition of autophagy. The results imply that ERα protected against BNIP3-induced apoptosis and BNIP3-induced autophagy.

### 2.5. ERα Down-Regulated BNIP3 Expression

**Figure 5:**
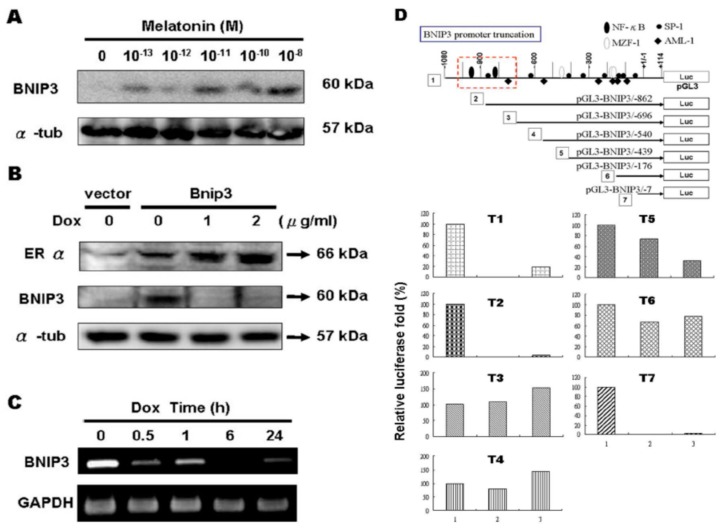
ERα/E2 inhibited BNIP3 mRNA and protein expression. (**A**) H9c2 cells were treated with indicated concentrations of melatonin in serum-free medium for 24 h. The level of BNIP3 protein increased in a concentration-dependent manner; (**B**) Tet-on ERα H9c2 cells were transfected with BNIP3 (6 μg), incubated for 6 h, and were then exposed to doxycycline (0, 1, 2 μg/mL) in serum-free medium for 18 h. Proteins were then extracted for Western blot analysis. The protein level of BNIP3 decreased in an ERα-dependent manner; (**C**) We found a time-dependent relationship between BNIP3 and ERα. The time course of exposure of doxycycline showed repression of BNIP3 mRNA transcripts; (**D**) Luciferase assay showed the exposure of ERα and/or E2 with pGL3-BNIP3 presented nearly 100% repression of BNIP3 luminescence (T1, T2 and T7). Bar 1: pGL3-T, Bar 2: pGL3-T+ERα, Bar 3: pGL3-T+ERα+E2, T1–T7: BNIP3 promoter truncation 1–7.

We also evaluated the expression of BNIP3 protein in cells that had been exposed to the ERα inhibitor melatonin (10–13~10–8 M). The results revealed a concentration-dependent increase in the expression of the BNIP3 protein (Figure 5A). Furthermore, there was a dose-response relationship between the amount of doxycycline administered and the level of BNIP3 protein expression in Tet-on ERα H9c2 cells (Figure 5B). The results suggest that ERα interferes with the expression of BNIP3 proteins. In addition, RT-PCR analysis revealed that BNIP3 mRNA expression was attenuated by doxycycline treatment in a time-dependent manner (Figure 5C). Analysis of the ERα regulatory sites of the BNIP3 promoter using luciferase reporter plasmids (pGL3) containing different truncations of the BNIP3 promoter showed that the relative luciferase activity of T1, T2 and T7 was greatly decreased in cells exposed to ERα and E2 (Figure 5D). Based on those findings, it appears that either NFκB or SP1 serves as a binding site for ERα and that ERα inhibits the gene expression of BNIP3.

## 3. Discussion

The processes of autophagy and apoptosis are evidently associated with the pathological effects of various cardiac disease conditions such as dilated cardiomyopathy, aortic stenosis, and starvation [2,44,45,46,47]. Development of autophagy involves the role of *ATG* genes that encode proteins needed for the induction of autophagy and the generation, maturation, and recycling of autophagosomes [48]. Beclin-1, also known as Atg6, mediates the recruitment and localization of other Atg proteins to the phagophore and plays an important role in autophagosome formation [2,49]. Moreover, the formation of autophagosomes requires Atg5-Atg12 and LC3 (Atg8)-phosphatidyl ethanolamine (PE) conjugation systems [1,2]. The Microtubule-associated protein light chain-3 (LC3) is cleaved by cysteine protease Atg4 to LC3-I that conjugates with PE to generate LC3-II and associates with the autophagosome membrane. The level of LC3-II is therefore often used as an autophagy marker in molecular studies [1,50]. In addition, autophagy is regulated by the mammalian target of rapamycin complex 1 (mTORC1), which is activated by Akt signaling and inactivated by adenosine monophosphate-activated protein kinase (AMPK) signaling [51].

Beclin-1 and the members of Bcl-2 family serve as a point of crosstalk between the autophagic and apoptotic pathways [52,53]. Beclin-1 can inactivate the autophagic process during its interaction with the anti-apoptotic proteins Bcl-2 and Bcl-XL [54,55]. BNIP3 can also induce autophagy by binding to Rheb, an mTOR activator, thereby blocking mTOR activation [14]. BNIP3 is involved in the activation of Beclin-1 by competing with Beclin-1 for binding to Bcl-2/Bcl-XL resulting in the induction of autophagy. Moreover, apoptosis in cardiomyocytes is attributed to caspase activation through extrinsic and intrinsic signaling pathways [56,57]. Caspase 3 on cleavage activation acts as one of the major effectors of apoptosis [58].

In this study, overexpression of BNIP3 triggered substantial mitochondrial damage in the H9c2 cardiomyoblasts. BNIP3-induced mitochondrial damage contributed to a significant increase in the number of apoptotic nuclei correlated with an increase in the activation of caspase 3. BNIP3 is also known to trigger necrosis through mitochondrial permeability transition pore formation with characteristic early loss of plasma membrane integrity and ATP [59]. Therefore, BNIP3 may also activate caspase independent necrosis like the cell death pathway. However, the present study shows that BNIP3 induces apoptosis in a caspase-dependent manner which is in accordance with our previous studies on hypoxia-associated effects on H9c2 cells as well as in neonatal rat ventricular myocytes (NRVMs) [60,61,62,63,64]. Estrogen metabolites are known to modulate the expression of the hypoxia associated with Hif1α [65,66]. Administration of 2-Methoxyestradiol, an inhibitor of Hif1α, has been shown to provide neuro-protection against cerebral ischemia and traumatic brain injury in animal models. Inhibition of Hif1α dependent BNIP3 levels by 2-Methoxyestradiol is known to attenuate cellular apoptosis and provide neuro protection against traumatic brain injury [65]. In our observation in heart cells, along with the mitochondrial death signaling, BNIP3 also induced an autophagy marker that has been shown to induce LV remodeling post myocardial infarction. Vande Velde et al. reported that BNIP3 induces a late DNA fragmentation after 24 h ATP [59]. Our results also show that BNIP3 over expression induces Beclin-1 expression only after 24 h and therefore the late apoptosis-induced BNIP3 correlated with the autophagic response with respect to initiation time.

Myocardial infarction that results from cardiac ischemic injury progresses to cardiac remodeling process [67]. Inhibition of cardiomyocyte apoptosis is an attractive therapeutic approach to protect the heart from associated post-infarction remodeling; however, a strategy that involves the inhibition of the activation of effectors such as caspase may cause systemic effects, which limits their application [28,29]. However, targeting the upstream mediators of apoptosis specific to ischemia-induced cardiomyocyte death is a viable alternative strategy [31,49,68]. In this context, BNIP3, which is upregulated via HIFα during hypoxia in the heart and other tissues, is an ideal target [13,30,31].

Although apoptosis is a well-established consequence of hypoxia-induced myocardial injury, few studies have evaluated the role that autophagy plays in cells under hypoxic conditions. A previous study showed that under conditions such as hypoxia, the survival signaling pathway in heart cells is down-regulated and FOXO3a-induced BNIP3 expression contributes to increased autophagy and apoptosis [60]. In this study, overexpression of BNIP3 induced both apoptosis and autophagy in cardiomyocytes. An increase in the incidence of the autophagic process was due to the elevated levels of BNIP3-induced Beclin-1. BNIP3-associated mitochondrial death and mitophagy has been shown to cause LV remodeling post myocardial infarction, and targeting/inactive BNIP3 has been a tested rationale in restraining ischemic cardiomyocytes from apoptosis and in cardioprotection against systolic heart failure [13,69,70,71,72]. However, the cardio-protective effects of ERα in an ideal in vivo model against BNIP3-induced cell death are not well understood yet. Further, an understanding of the effects of ERα overexpression on ERβ is not yet substantiated and requires further investigation.

In the previous few decades, several reports have shown protective effects of hormone replacement therapy in menopausal women against ischemic heart disease, coronary heart disease, [73,74,75]. E2 replacement has also shown effective protection against psychological stress seen in estrogen-replaced ovariectomized rats, and different ERs have also been proven to confer cardio-protection [35,76,77]. Hale et al. reported that estrogen replacement reduces both myocardial infarct size and ventricular arrhythmias induced by ischemia/reperfusion in both female and male rabbits [78]. Numerous studies have provided substantial evidence to show that ERα protects the heart from hypertrophy, aging and ischemia reperfusion injury [33,34,79,80]. In this study, we further show that ERα/E2 plays a cytoprotective role by protecting against BNIP3-induced apoptosis and autophagy.

According to the results, overexpression of ERα and administration of E2 inhibited the apoptotic or autophagic responses triggered by BNIP3 overexpression in H9c2 cells. Further analysis showed that ERα interferes with BNIP3 binding to the mitochondrial membrane and simultaneously interferes with BNIP3 expression by downregulating BNIP3 promoter activity. Based on the findings, we suggest that ERα plays a cytoprotective role by retarding the expression of BNIP3 in cardiomyocytes. Our results further indicate that ERα interacts with BNIP3 to restrain BNIP3 from activating cell death. Further, the expression of BNIP3 was also attenuated by the transcriptional inactivation level in cells that were exposed to ERα.

## 4. Material and Methods

### 4.1. Cell Culture and Transfection

Cell culture was performed following the methods given in our previous publications [35]. BD1X Rat embryonal heart H9c2 cells (CRL-1446, ATCC, Manassas, VA, USA) were grown in DMEM (D2906, Sigma-Aldrich, St. Louis, MO, USA), supplemented with 10% FBS and 1.5 g/L sodium bicarbonate, and then incubated at 37 °C in a 5% CO_2_ incubator. Full lengths of the BNIP3 open reading frame were cloned and inserted into the *Bam*HI site of pcDNA3-HA for BNIP3 protein expression. All plasmids were prepared using the AxyPrepTM Plasmid Maxiprep kit (Axygen, Inc., Union City, CA, USA) and were transfected into cells using GeneJuice^®^ (Novagen, Merck, Darmstadt, Germany) transfection reagent according to the manufacturer’s guidelines. After 6 h, the cells were fed with fresh medium followed by drug treatment.

### 4.2. Western Blot Analysis

Western blot analysis of protein expression was performed following previous reports with slight modification [81]. Proteins in cell lysates were separated using SDS-PAGE and transferred to PVDF membranes (GE life sciences, Marlborough, MA, USA). Residual protein sites were blocked in Tween/Tris-buffer saline (TBS) containing 5% skim milk. The filters were incubated with diluted primary antibodies in TBS plus 5% skim milk at the recommended concentrations at 4 °C overnight and incubated with secondary antibodies for 1 h at room temperature. Antibody reaction was visualized with ECL reagent (Merk Millipore, Burlington, MA, USA).

### 4.3. DNA Fragmentation

Cells were grown to 5 × 10^6^ confluence and then harvested using a tabletop microcentrifuge at maximum speed for 10 s. A 50-μL aliquot of lysis buffer (50 mM Tris-HCl (pH 7.4), 20 mM EDTA, and 1% IGEPAL-630 was added and then the mixture was centrifuged at 15,000 rpm for 15 min. The supernatant (50 μL) was collected in a new tube, at which time 1% SDS and RNase A (final concentration 5 mg/mL) were added and allowed to incubate for 2 h at 56 °C. Proteinase K (final concentration 2.5 mg/mL) was then added and the mixture was allowed to incubate for 2 h at 37 °C, at which time 0.5 volume 3 M ammonium acetate and 2.5 volume ethanol were added to precipitate DNA for 1 h at −70 °C. Samples were centrifuged at 15,000 rpm for 15 min to collect DNA and the pellets were air-dried. DNA pellets were dissolved in 30 μL ddH2O, subjected to 1.5% agarose gel electrophoresis, and then visualized using the Kodak Scientific Imaging System, (Rochtester, NY, USA).

### 4.4. TdT-Mediated dUTP Nick End Labeling (TUNEL)

Air-dried cell samples were fixed with a freshly prepared fixation solution (4% paraformaldehyde in PBS, pH 7.4) for 1 h at room temperature. After a rinse with phosphate buffered saline (PBS), samples were incubated with blocking solution (3% H_2_O_2_ in methanol) for 10 min at room temperature. Samples were then rinsed with PBS and incubated in freshly prepared permeabilisation solution (0.1% Triton X-100 in 0.1% sodium citrate) for 2 min on ice. Cells were then rinsed twice with PBS, exposed to 50 μL TUNEL reaction mixture (Roche Diagnostics, Indianapolis, IN), covered with parafilm, and then incubated for 1 h at 37 °C in a humidified atmosphere in the dark. Samples were then rinsed 3 times with PBS and stained with DAPI diluted solution for 30 min at room temperature in the dark. Finally, cells were rinsed 3 times with PBS and then analyzed under a fluorescence microscope (Olympus DP73, Tokyo, Japan) in a detection range of 515–565 nm (green).

### 4.5. RT-PCR

An aliquot of total RNA (0.5 μg) was reverse transcribed using 0.5 μM oligo dT primers in a reaction solution (50 μL) containing 75 mM KCl, 50 mM Tris—HCl (pH 8.3), 3 mM MgCl_2_, 10 mM DTT, 10 units RNase inhibitor, 0.8 mM total dNTPs, and 200 units of MMLV reverse transcriptase. The sample was incubated at 42 °C for 1 h and at 99 °C for 5 min before chilling on ice for 10 min. The RT product (2 μL) was diluted with the PCR buffer (50 mM KCl, 10 mM Tris HCl, 2 mM MgCl_2_) to a final volume of 50 μL, containing 0.5 μM dNTPs (final concentration, 0.8 mM) and 0.5 units of Taq DNA polymerase. Following the hot start (5 min at 95 °C); the samples were subjected to 30 cycles of 1 min at 95 °C, 30 s at annealing temperature, and 1 min at 72 °C. After 30 cycles, the final cycle was extended for 10 min at 72 °C, and held at 4 °C. The PCR products were analyzed by 1.2% agarose gel electrophoresis, and imaged using the Kodak Scientific ID Imaging System.

### 4.6. Luciferase Assay

BNIP3 reporter constructs were provided by Professor Ching Li (National Chiayi University). Cells were transfected with 0.5 μg of plasmid DNA using GeneJuice^®^ transfection reagent according to the manufacturer’s protocol. After 24 h, cells were harvested for luciferase assay using a Dual-Luciferase Report Assay System (Promega, Madison, WI, USA). In brief, cell lysates were prepared by adding 1× passive lysis buffer and then shaken for 15 min. A 100-μL aliquot of Luciferase assay Reagent II (LAR II) was added to 96-well plates, followed by the addition of 20 μL of lysate. The reagent and the lysate were thoroughly mixed by pipetting gently, and luciferase activity was measured using a luminometer that had been programmed for a 2-s measurement delay followed by a 1-s measurement reading. Stop & Glo reagent was then added to detect cells expressing Renilla luciferase luminescence. Firefly luminescence data were normalized to data of Renilla luminescence.

### 4.7. Statistical Analysis

Each experiment was repeated at least three times, and the results are expressed as the mean ± SEM. Statistical comparisons were made using the Student’s *t*-test. A *p*-value < 0.05 was considered to represent statistical significance.

## Supplementary Materials

Supplementary materials can be found at http://www.mdpi.com/1422-0067/19/5/1298/s1.
