# Supplementary material for: Estrogen and/or Estrogen Receptor α Inhibits BNIP3-Induced Apoptosis and Autophagy in H9c2 Cardiomyoblast Cells
Source: Int J Mol Sci. 2018 Apr 26;19(5):1298. doi: 10.3390/ijms19051298 (PMC5983791; doi:10.3390/ijms19051298)
Supplement: Supplementary file 1 [file ijms-19-01298-s001.pdf]

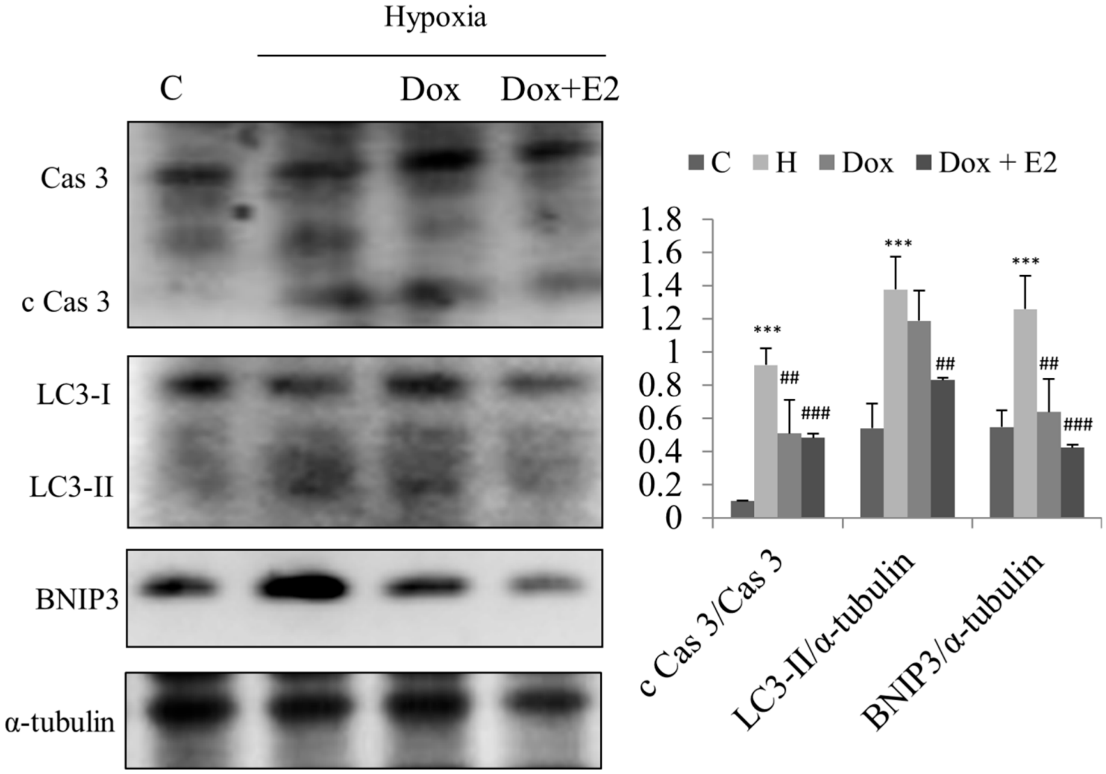

Supplementary Figure 1. Effects of Estrogen and Estrogen Receptor on hypoxia induced Caspase 3 activation and LC3-II levels. Representative western blots show the levels of caspase 3, LC3 and BNIP3 in control neonatal rat ventricular myocytes (NRVMs) (C), Hypoxia treated NRVMs, Dox induced ERα expressing NRVMs under hypoxia (Dox), Dox induced ERα expressing NRVMs with Estrogen treatment under hypoxia (Dox + E2). $n = 3$, ***$p < 0.001$ show significant difference with respect to control NRVMs. group; ##$p <$ 0.01 and ###$p < 0.001$ show significant difference with respect to control hypoxia group.

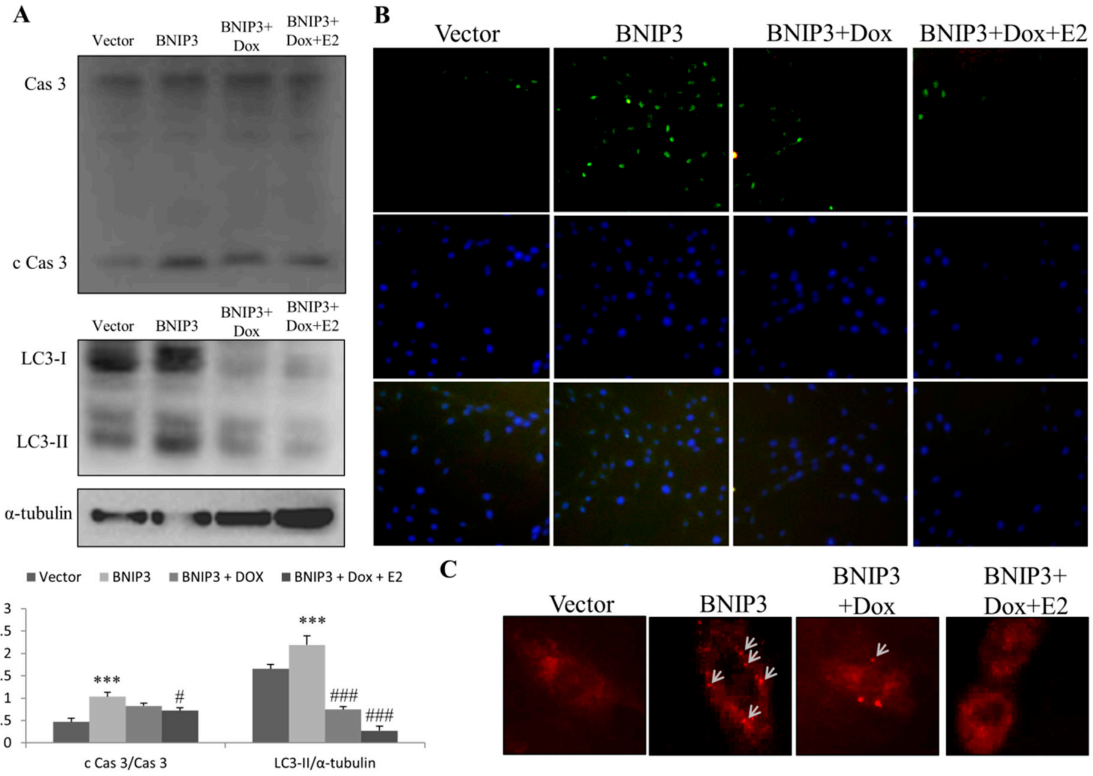

Supplementary Figure 2. Effects of Estrogen and Estrogen Receptor on BNIP3 induced Caspase 3 activation and LC3-II levels. A, Representative western blots show the levels of caspase 3 and LC3 and BNIP3 in control neonatal rat ventricular myocytes (NRVMs) transfected with empty vector (Vector), BNIP3 overexpressed NRVMs (BNIP3), Dox induced ERα expressing NRVMs with BNIP3 over expression (BNIP3 + Dox), Dox induced ERα expressing NRVMs with Estrogen treatment and BNIP3 over expression (BNIP3 + DOX + E2). B, Apoptosis levels detected by Tunel assay. C, LC3 puncta assay to show autophagy levels. White arrows show the LC3 puncta formation in the cells undergoing autophagy $n = 3$, ***$p < 0.001$ show significant difference with respect to control NRVMs. group; #$p < 0.05$ and ###$p < 0.001$ show significant difference with respect to control BNIP3 overexpression group.
